# Technology access, use, socioeconomic status, and healthcare disparities among African Americans in the US

**DOI:** 10.3389/fpubh.2025.1547189

**Published:** 2025-05-28

**Authors:** Ebenezer Larnyo, Sharon Tettegah, Jonathan Aseye Nutakor, Stephen Addai-Dansoh, Francisca Arboh

**Affiliations:** ^1^Center for Black Studies Research, University of California, Santa Barbara, Santa Barbara, CA, United States; ^2^College of Creative Studies, University of California, Santa Barbara, Santa Barbara, CA, United States; ^3^Department of Health Policy and Management, Jiangsu University School of Management, Zhenjiang, Jiangsu, China; ^4^Teesside International Business School, Teesside University, Middlesbrough, United Kingdom

**Keywords:** technology access, healthcare technology use, technology inequity, socioeconomic status, healthcare disparity/health disparities, African American

## Abstract

**Background:**

Healthcare disparities remain a significant challenge in addressing equitable healthcare access and outcomes for minority populations, including African Americans. Rooted in systemic racism and historical exclusion, these inequities persist as part of broader structural violence. Leveraging health technology holds promise in addressing these disparities by enhancing access to care, improving its quality, and reducing inequities. However, the association between health technology access, use, socioeconomic status (SES), and healthcare disparities among African Americans remains underexplored. This study aims to explore the potential role of technology in mitigating healthcare disparities by investigating the associations between technology access, healthcare technology use, socioeconomic status (SES), and health disparities among African Americans.

**Methods:**

Using data from the Health Information National Trends Survey (HINTS) Wave 6 dataset, a sample of 815 African Americans was analyzed using Partial Least Squares-Structural Equation Modeling (PLS-SEM).

**Findings:**

The results of the study showed that technology access had a significant positive effect on healthcare technology use (*β* = 0.260, *p* < 0.000). Technology access (*β* = −0.086, *p* = 0.034) and healthcare technology use (*β* = −0.180, *p* < 0.001) demonstrated a significant negative effect on healthcare disparity, respectively. Results also revealed SES had a significant positive effect on technology access (*β* = 0.424, *p* < 0.001). Additionally, SES was found to significantly moderate the relationship between technology access and healthcare disparities, indicating variability in the impact of technology access based on SES levels among African Americans.

**Conclusion:**

These findings highlight the potential of technology in mitigating healthcare disparities among African Americans. By promoting enhanced health technology access and utilization, particularly in lower SES populations, the healthcare outcomes for vulnerable communities can be significantly improved. Policymakers, healthcare providers, and technology developers are encouraged to collaborate in providing conducive conditions for the adoption and use of technology to advance healthcare equity.

## Introduction

In recent years, advancements in technology have transformed various aspects of society, including the healthcare sector. These advancements have revolutionized healthcare delivery, enabling access to improved personalized healthcare, enhanced patient outcomes, and more efficient care systems (1, 2). However, despite these advancements, significant disparities in healthcare outcomes persist among marginalized communities, particularly African Americans (3–7). The Centers for Disease Control and Prevention (CDC) defines health disparities as preventable differences in the burden of disease, injury, violence, or opportunities to achieve optimal health that are experienced by socially disadvantaged populations (8). Technology inequity, characterized by unequal access to and utilization of healthcare technologies, has emerged as a critical factor contributing to these disparities (9).

Healthcare inequities faced by African Americans are not incidental but rooted in systemic racism and historical patterns of exclusion (3, 5, 10). Saidiya Hartman’s *‘Afterlife of Slavery’* highlights how skewed life chances, by extension technological exclusion, and healthcare disparities are enduring legacies of slavery and racial capitalism (11). Similarly, Ruth Wilson Gilmore’s framing of racism as *‘the production of premature death’* (12) underscores how systemic barriers, such as limited access to telehealth, electronic health records, and wearable technologies, disproportionately deny Black populations equitable opportunities for healthcare (13). While efforts have been made to address these disparities, the rapid pace of technological advancements has introduced a new dimension to the problem (14, 15). Technology inequity exacerbates healthcare disparities among African Americans, as they often encounter limited access to essential technologies, such as electronic health records (EHRs), telehealth services, mobile health applications, and wearable devices, and also experience delayed access to needed care, and discrimination (16, 17).

To comprehend the multifaceted impact of technology on healthcare disparities among African Americans, it is crucial to explore the underlying factors that perpetuate this divide. Socioeconomic factors, including income disparities, education, occupation, and lack of health insurance coverage, play a significant role in limiting access to and utilization of healthcare technologies (14). Low socioeconomic status (SES) individuals, including many African Americans, often face barriers to technology access due to limited financial resources, lower digital literacy levels, and inadequate technology infrastructure in their communities. Additionally, continued systemic discrimination (18, 19), delayed access to needed care (20, 21), and health risk factors (18) may contribute to the divide, further impeding African Americans’ ability to leverage technology for improved health outcomes.

Several studies have demonstrated the detrimental effects of technology inequity on healthcare disparities (22) among African Americans (23–26). Research by Mulia et al. indicated that Hispanic/Latinx and African American patients had reduced access to telehealth services compared to their white counterparts, resulting in delayed or inadequate healthcare access (27). Furthermore, Heiney et al. found that African Americans faced barriers to utilizing mobile health applications, limiting their ability to engage in self-management and preventive care practices (28).

This study aims to comprehensively explore the complex relationship between technology inequity and healthcare disparities among African Americans. By analyzing data from the Health Information National Trends Survey (HINTS) Wave 6 dataset, we seek to identify the underlying mechanisms through which technology inequity perpetuates healthcare disparities. Furthermore, we discuss potential strategies and interventions to address these disparities and promote equitable access to healthcare technologies for African Americans.

## Methodology

### Study population and design

Using data from the National Cancer Institute (NCI)’s Health Information National Trends Survey (HINTS) Wave 6 dataset, this study analyzed responses from 815 African Americans. HINTS 6 is a cross-sectional survey of non-institutionalized civilian adults aged 18+ years in the United States collected from March 7, 2022, to November 8th, 2022 with the goal of investigating the need for, access to, and use of health-related information and health-related behaviors, perceptions and knowledge (29, 30).

HINTS 6 included an embedded methodological experiment comparing two mixed-mode approaches: concurrent and sequential; also referred to as the control and treatment groups, respectively. Households in the control group received a cover letter with the link to the web survey and their unique access code as well as a paper survey with each mailing (including their first mailing). Households in the treatment group received only a cover letter with the link to the web survey and their unique access code with their first mailing, they did not receive a paper survey in their first mailing. In subsequent mailings, these households received the link to the web survey and their unique access code as well as the paper survey. Both conditions used the same sampling frame provided by Marketing Systems Group (MSG) of addresses in the United States. All addresses were grouped into one of four strata; high and low minority, and by rural and urban area using the benchmark for assessing minority populations in census data. This approach enhances the representativeness of minority populations, including African Americans, ensuring sufficient representation of diverse demographic segments and thus facilitating accurate generalization to the African American population in the U.S.

The mailing protocol for HINTS 6 followed a modified Dillman approach (31) with all selected households receiving a total of four mailings: an initial mailing, a reminder postcard, and two follow-up mailings.

### Measures

#### Health disparity (H_DISPARITY)

The outcome variable for this study was health disparity. To assess health disparity, the experiences of discrimination while seeking medical care and delays in accessing necessary care were used. To evaluate delay in accessing needed healthcare, a 3-item scale of “Yes,” “No, I received the medical care I felt I needed” and “I did not need any medical care in the past 12 months,” was used to elicit response to the question “In the past 12 months, did you delay or not get medical care you felt you needed - such as seeing a doctor, a specialist, or other health professional?.” Discrimination was evaluated on a yes/no scale on a question “Have you ever been treated unfairly or been discriminated against when getting medical care because of your race or ethnicity?”

### Independent variables

#### Socioeconomic status (SES)

The relationship between socioeconomic status (SES), technology access, and healthcare disparities is complex and multifaceted. SES, which encompasses factors such as income, education, and occupation, plays a significant role in determining an individual’s access to technology and subsequent healthcare disparities. Many studies have established a strong relationship between socioeconomic factors and how they influence an individual’s access to technology (32, 33). Higher-income individuals are more likely to afford smartphones, computers, and internet access, which are essential for utilizing healthcare technology such as telehealth services or health apps (34). On the other hand, lower-income individuals may face barriers due to the cost of devices, internet access, or limited availability of technology infrastructure in their communities (35). Consequently, disparities in technology access may contribute to disparities in healthcare outcomes. Furthermore, the digital divide, driven by socioeconomic factors, exacerbates healthcare disparities. For instance, lower-income individuals, including African Americans who are more likely to experience economic challenges, may lack the resources or skills to effectively use health technology. This limits their ability to access online health information, engage in telehealth visits, or effectively manage their healthcare. As a result, they may experience delays in accessing care, receive suboptimal healthcare services, or have poorer health outcomes compared to individuals with higher SES (14, 36). SES is closely linked to health literacy, which refers to an individual’s ability to access, understand, and use health information to make informed decisions about their health (37, 38). Individuals with lower SES are more likely to have lower health literacy levels, which can hinder their utilization of healthcare technology. Limited health literacy skills may make it difficult for individuals to navigate complex digital platforms, understand health-related information, or effectively communicate with healthcare providers through technology (37, 38). Another factor that could be influenced by SES is provider-patient communication. Technology-mediated interactions, such as telehealth visits or patient portals, may impact provider-patient communication differently based on SES. Individuals with lower SES may face challenges in effectively communicating their health concerns, understanding medical jargon, or asking necessary questions through these platforms. These communication barriers can impede the delivery of patient-centered care and contribute to disparities in healthcare quality and outcomes (39). Education and technological literacy have the potential to influence the level of SES of an individual (33). Education, another component of SES, influences technological literacy and digital skills. Higher levels of education are associated with better technology proficiency (33, 40, 41), including the ability to navigate digital platforms, access online health resources, and use health technology effectively. Individuals with lower education levels may experience difficulties in adopting and benefiting from healthcare technology, leading to disparities in healthcare access, utilization, and health outcomes (42, 43). Thus, it is imperative to understand how SES plays into technology access and use and how they consequently impact healthcare disparity among African Americans. Respondents’ education level and household income were used to evaluate socioeconomic status. For education, respondents were asked about their highest level of education. The highest level of education was categorized as “Less than high school,” “high school graduate,” “some college,” “bachelor’s degree,” and “post-baccalaureate degree.” Similarly, respondents’ annual household income was assessed with 1 representing “$0 to $9,999”, 2 “$10,000 to $14,999”, 3 “$15,000 to $19,999”, 4 “$20,000 to $34,999”, 5 “$35,000 to $49,999”, 6 “$50,000 to $74,999”, 7 “$75,000 to $99,999”, 8 “$100,000 to $199,999” and 9 as “$200,000 or more”.

Based on the above, we posit the following hypothesis for SES:

**H1:** SES has a significant negative effect on health disparity; **H2:** SES has a significant positive effect on technology access, and **H6:** SES moderates the relationship between technology access and health disparity, such that the higher an individual’s SES level, the lesser their experience of healthcare disparity.

#### Technology access (TECH_ACC)

Technology and its associated advancements have long been trumpeted to influence the kind of healthcare individuals receive and the quality of such care (44). Nonetheless, through what has been known as the “Digital Divide,” technology might either assist in making things better or perhaps worse. For some populations, having access to digital information might help with self-care and maintaining good health (32). However, the racial and ethnic groups that experience the largest injustices, particularly Blacks and Hispanics, have significantly varying access to digital resources depending on socioeconomic level. The future will depend on improving digital health equality. Broadband access represents one specific major concern. Over 21 million Americans lack broadband access (45). While cities like New York have broadband infrastructure covering 99.9% of the population, 2.2 million adults there do not have a home broadband subscription (46). In more rural areas, such as the mountains of Appalachia in states such as Tennessee, Kentucky, and West Virginia, there are large areas with no or limited broadband access (46). According to the Public Policy Institute of California (PPIC), though broadband has grown slightly from 84% in 2019 to 85% in 2020, racial and ethnic disparities in access persist with 81% of Latino, 83% of Black, 87% of white, and 88% of Asian households report having broadband access at home in 2021 (33). Thus, disparities in broadband infrastructure, driven by uneven geographical deployment, economic affordability issues, and historical digital redlining, constitute critical barriers that disproportionately affect marginalized communities, including African Americans. Aside from broadband access, telehealth access is critically essential in promoting equitable healthcare. The COVID-19 pandemic has facilitated and increased the importance and use of telehealth (46). Because of the risk of person-to-person viral transmission, organizations around the country switched most outpatient care to telehealth essentially overnight (46). Black patients and poorer patients were much more likely to receive telephonic as opposed to video visits (46). The following questions were posed to respondents to measure technology access to access to basic cell phones, smartphones, and access and use of the internet rated on a “Yes”/“No” scale; (i) “Have a basic cell phone?,” (ii) “Have a smartphone?” and (iii) “Do you ever go online to access the Internet or World Wide Web, or to send and receive email?.” Based on the above, this study posits that: **H3:** Technology access has a significant negative effect on healthcare disparities, **H4:** Technology access has a significant positive effect on health technology, and **H7:** There is a significant negative indirect effect of SES on H_DISPARITY through the sequential mediation of TECH_ACC.

#### Health technology use (hTECH_USE)

Health technology has the potential to significantly impact healthcare outcomes for African Americans, as it may help improve access to care, enhance patient engagement, and promote health equity. For instance, during the COVID-19 pandemic the use of telehealth became more prevalent, helping individuals manage their chronic diseases by enhancing patient-provider communication, promoting medication adherence, and enabling self-monitoring of health conditions (47, 48). However, it is crucial to acknowledge that disparities in health technology use may also contribute to existing healthcare disparities (49), particularly issues of access due to structural and systemic racism. Thus, to measure the health technology use among African Americans, this study evaluated the frequency of watching a health-related video on a social media site like YouTube in the last 12 months. The responses were assessed on a 5-likert scale with 1 as “Almost every day,” 3 as “A few times a month” and 5 as “Never.” Respondents were further asked how often they interacted with people who have similar health or medical issues as them on social media or online forums and how often they shared general health-related information on social media for example, a news article in the last 12 months, respectively. Finally, respondents were asked to indicate whether they had received care from a doctor or health professional using telehealth in the past 12 months using a scale of 1–4, with 1 indicating Yes, by video, 2 as “Yes, by phone call (voice only with no video),” 3 as “Yes, some by video and some by phone call” and 4 as “No telehealth visits in the past 12 months.”

These questions were posed to help understand how health technology use affects healthcare disparities, hence, the study posits that: **H5:** *Health technology use has a significant negative effect on healthcare disparities.*

**H8:** *There is a significant negative indirect effect of TECH_ACC on H_DISPARITY through the sequential mediation of hTECH_USE.*

### Statistical analysis

Due to its robustness of estimations and statistical power (50, 51), the partial least squares (PLS) based on structural equation modeling (SEM) was employed to test and validate the hypothesized model with the aid of SmartPLS 4.0 (52). The model’s validity was determined by analyzing the measurement and structural models.

The measurement model was evaluated using internal consistency reliability, convergent validity, and discriminant validity. This was done using the outer loadings (≥0.70 for reliability), Average of Variance Extracted (AVE; ≥0.50 indicating convergent validity), Composite Reliability (CR; ≥0.70 indicating internal consistency), Fornell and Larcker criterion (square root of AVE exceeding inter-construct correlations indicating discriminant validity), and Heterotrait-Monotrait Ratio of correlation (HTMT; <0.85 demonstrating discriminant validity) (53–55).

The collinearity assessment (VIF) (53, 54), path coefficient (*β*; showing relationship strength and direction), *t*-statistics (statistical significance) (53), and the model fit index were used to evaluate the structural model for the main model, mediation, and moderation analysis. The moderating effect was further evaluated using the simple slope analysis. Model fit analysis was performed using the Standardized Root Mean Square Residual (SRMR ≤0.10 acceptable), discrepancy function (d_ULS and d_G), chi-square statistic, and the Normed Fit Index (NFI) (56, 57).

## Results

Table 1 shows the summary statistics for the respondents’ demographics. The majority of respondents were females, and the most occurring age was 50–64 years old. Most respondents had some college education, with the majority having only one employment. Lastly, the most observed household income range was between $20,000 and $34,999.

**Table 1:** Demographic characteristics.

Item	Frequency	Percentage
Gender		
Male	253	31.04
Female	562	68.96
Age (in Years)		
18–34	109	13.37
35–49	152	18.65
50–64	287	35.21
65–74	191	23.44
75+	76	9.33
Marital status		
Married	230	28.22
Living as married or living with a romantic partner	40	4.91
Divorced	170	20.86
Widowed	92	11.29
Separated	31	3.80
Single	252	30.92
Education		
Less than High School	129	15.83
High School Graduate	194	23.80
Some College	301	36.93
Bachelor’s Degree	145	17.79
Post-Baccalaureate Degree	46	5.64
Occupational category		
Employed only	386	47.36
Homemaker only	12	1.47
Student only	10	1.23
Retired only	210	25.77
Disabled only	74	9.08
Multiple Occupation statuses selected	83	10.18
Unemployed for 1 year or more only	21	2.58
Unemployed for less than 1 year only	14	1.72
Other Occupation only	5	0.61
Income range		
$0 to $9,999	25	3.07
$10,000 to $14,999	113	13.87
$15,000 to $19,999	88	10.80
$20,000 to $34,999	156	19.14
$35,000 to $49,999	108	13.25
$50,000 to $74,999	117	14.36
$75,000 to $99,999	47	5.77
$100,000 to $199,999	72	8.83
$200,000 or more	89	10.92

Due to rounding errors, column-wise percentages may not equal 100%.

**Figure 1:**
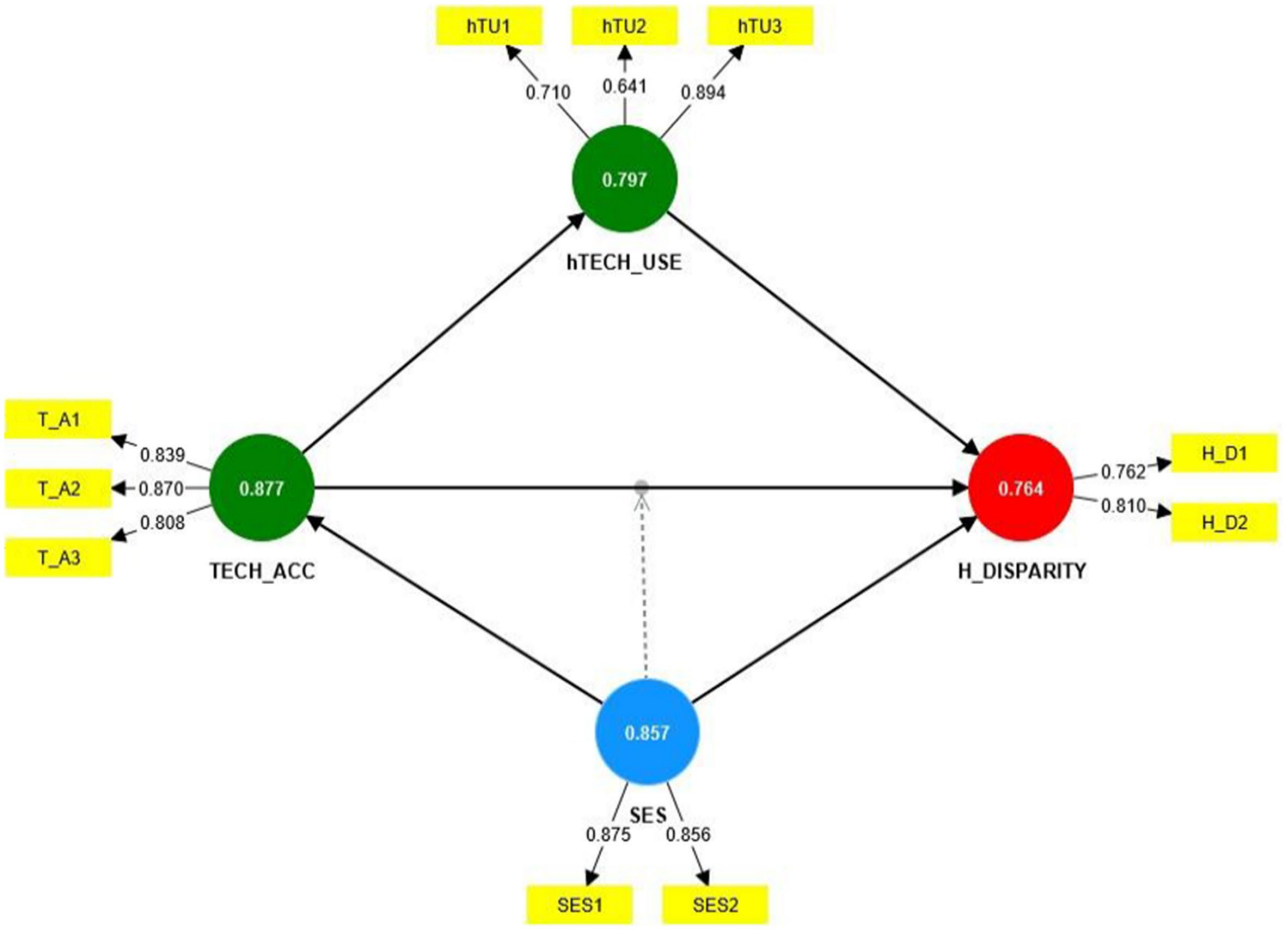
Path diagram showing loadings and composite reliability of the various latent and observed variables.

The results for the outer loadings, internal consistency reliability, convergent validity, and discriminant validity are presented in Table 2 above. The outer loadings for all constructs showed values greater than the acceptable threshold of 0.70, except hTU2, which had an outer loading value of 0.694. Also, composite reliability values for all the constructs were greater than 0.70, suggesting strong internal consistency reliability (38). AVE values were all above the recommended level of 0.50 (55), as shown in Table 2. Figure 1 shows the various constructs with their respective loadings. Results of HTMT showed all the constructs had HTMT values less than the threshold of 1. Additionally, the square root of the AVE of all the constructs was greater than their correlation with other constructs, and the diagonal items were larger than the entries in corresponding columns and rows, hence satisfying the Fornell and Larcker criterion (55).

**Table 2:** Measurement model.

Constructs	Notation	Convergent validity		Internal consistency reliability
		Outer loading	AVE	CR
Technology access	T_A1	0.839	0.705	0.877
	T_A2	0.870		
	T_A3	0.808		
Health technology use	hTU1	0.710	0.572	0.797
	hTU2	0.641		
	hTU3	0.894		
Socioeconomic status	SES1	0.875	0.750	0.857
	SES2	0.856		
Healthcare disparity	H_D1	0.762	0.619	0.764
	H_D2	0.810		

	H_DISPARITY	SES	TECH_ACC	hTECH_USE
Heterotrait-Monotrait Ratio (HTMT)—matrix				
H_DISPARITY				
SES	0.158			
TECH_ACC	0.142	0.572		
hTECH_USE	0.344	0.206	0.307	
Fornell-Larcker criterion				
H_DISPARITY	0.786			
SES	−0.061	0.866		
TECH_ACC	−0.065	0.424	0.839	
hTECH_USE	−0.183	0.135	0.260	0.756

H_DISPARITY, healthcare disparity; SES, socioeconomic status; TECH_ACC, technology access; hTECH_USE, healthcare technology use; AVE, Average Variance Extracted (≥0.50 recommended); CR, Composite Reliability (≥0.70 recommended); HTMT, Heterotrait-Monotrait Ratio (<0.85 recommended).

**Figure 2:**
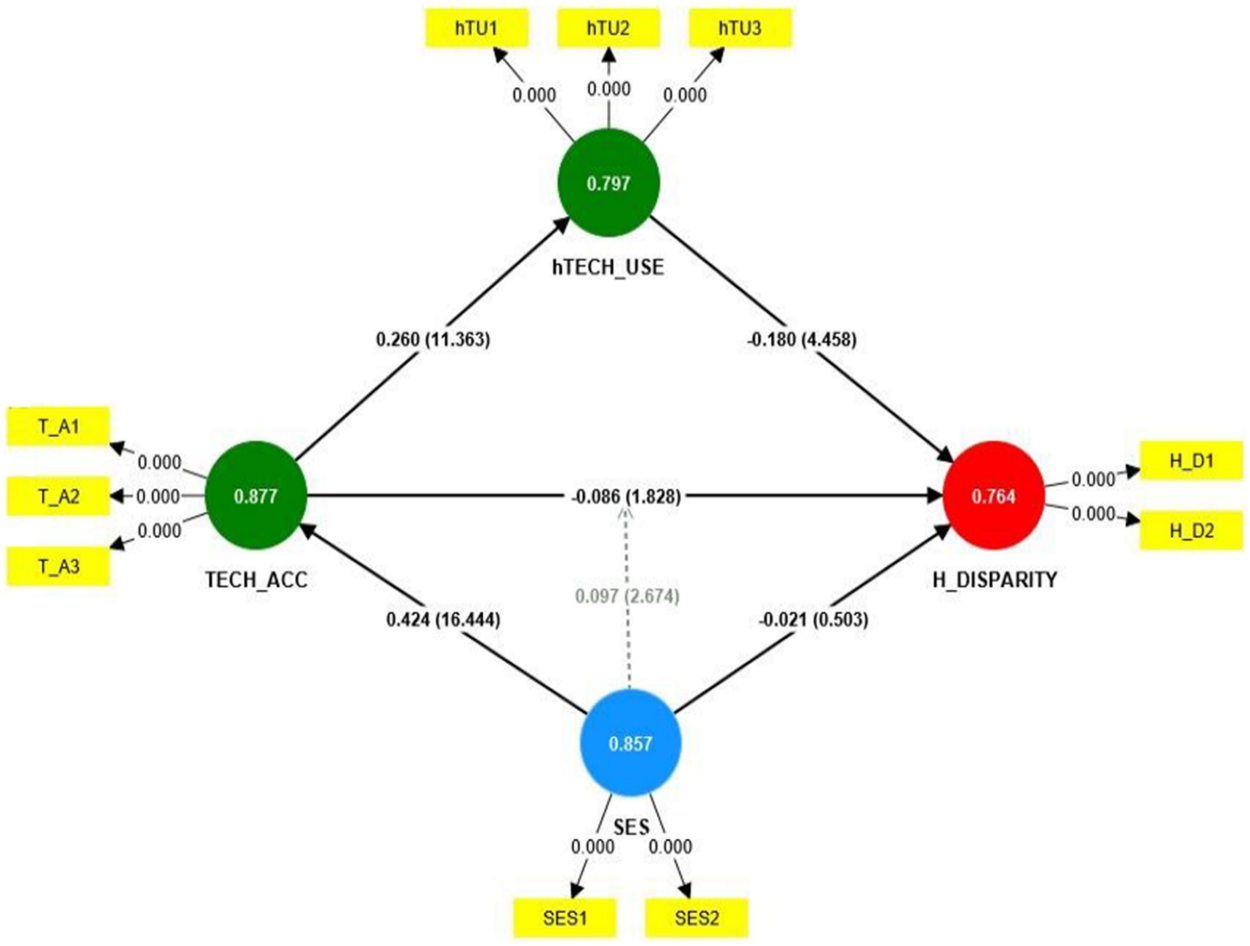
Path diagram showing bootstrapped results.

Results of the structural model as shown in Table 3 revealed a statistically significant relationship between SES → TECH_ACC (*β* = 0.424, *t*-statistics = 16.444, *p* < 0.001), TECH_ACC → H_DISPARITY (*β* = −0.086, *t*-statistics = 1.828, *p*-value = 0.034), TECH_ACC → hTECH_USE (*β* = 0.260, *t*-statistics = 11.363, *p* < 0.001), and hTECH_USE → H_DISPARITY (*β* = −0.180, *t*-statistics = 4.458, *p* < 0.001), thus, supporting hypothesis H2, H3, H4, and H5, respectively. However, SES → H_DISPARITY (*β* = −0.021, *t*-statistics = 0.503, *p*-value = 0.307) was not significant, therefore not supporting H1. Mediation results shows a partial mediation SES → TECH_ACC → H_DISPARITY (*β* = −0.036, *t*-statistics = 1.794, *p*-value = 0.036) and TECH_ACC → hTECH_USE → H_DISPARITY (*β* = −0.047, *t*-statistics = 4.332, *p* < 0.001), hence satisfying H7 and H8, respectively. The path diagram for the bootstrapped results is shown in Figure 2 below.

**Table 3:** Structural model assessment.

Hypothesis	Path	*β*	*t*-Statistics	*p* Value	Hypothesis supported or not
H1	SES → H_DISPARITY	−0.021	0.503	0.307	Not Supported
H2	SES → TECH_ACC	0.424	16.444	0.000	Supported
H3	TECH_ACC → H_DISPARITY	−0.086	1.828	0.034	Supported
H4	TECH_ACC → hTECH_USE	0.260	11.363	0.000	Supported
H5	hTECH_USE → H_DISPARITY	−0.180	4.458	0.000	Supported
Moderating effect					
H6	SES x TECH_ACC → H_DISPARITY	0.097	2.674	0.004	Supported

Mediating effects					
Specific indirect effects					
Hypothesis	Path	*β*	*t*-Statistics	*p* Value	Remarks
H7	SES → TECH_ACC → H_DISPARITY	−0.036	1.794	0.036	Partial Mediation
H8	TECH_ACC → hTECH_USE → H_DISPARITY	−0.047	4.332	0.000	Partial Mediation

Model fit		
Fit Indices	Saturated model	Estimated model
SRMR	0.096	0.096
d_ULS	0.504	0.504
d_G	0.201	0.201
Chi-square	1,067.243	1,067.650
NFI	0.424	0.424

*β*, Path coefficients indicating relationship strength/direction; *t*-values >±1.65 and *p*-values <0.05 indicate significance. SRMR ≤0.10 indicates acceptable model fit.

**Figure 3:**
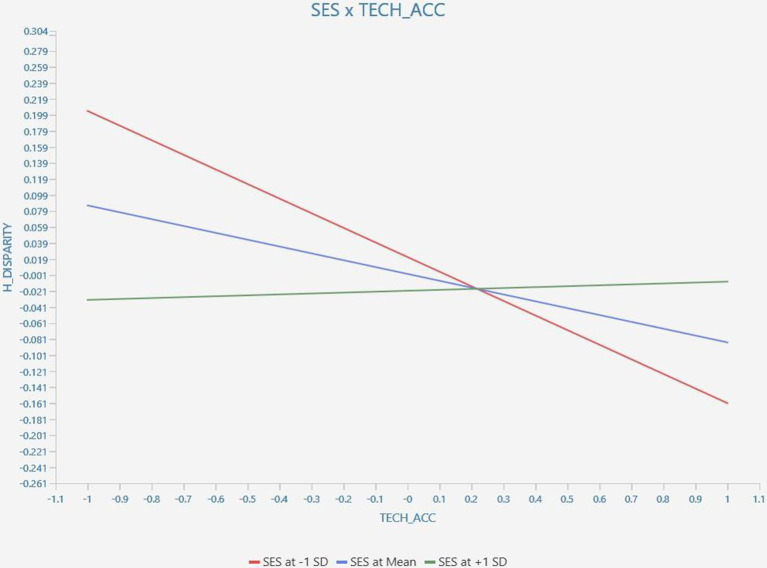
Result of the simple slope analysis of the moderating effect of SES on TECH_ACC → H_DISPARITY.

The moderating effect of SES x TECH_ACC → H_DISPARITY (*β* = 0.097, *t*-statistics = 2.674, *p*-value = 0.004) produced a statistically significant result, thus, satisfying H6. The simple slope analysis of the moderating effect is shown in Figure 3.

Finally, the summary of the fit indexes showed SRMR of 0.096, Unweighted Least Squares discrepancy (d_ULS) and Bentler’s Comparative Fit Index (d_G) values of 0.504 and 0.201, respectively, and NFI of 0.424, suggesting model fit.

## Discussion

This study aimed to investigate the relationships between socioeconomic status (SES), technology access, health technology use, and healthcare disparities. The findings revealed a positive association between SES and technology access, suggesting that individuals with higher socioeconomic status are more likely to have more access to technology. This aligns with previous research indicating that socioeconomic factors play a crucial role in determining technology access (32, 33, 58). The statistically significant relationship between SES and technology access highlights the role of structural racism in shaping economic opportunities and digital inclusion for African Americans. These disparities are not simply socioeconomic but are rooted in what Ruth Wilson Gilmore terms ‘group-differentiated vulnerability to premature death.’ (12) Communities with limited access to health technology face compounded disadvantages, reflecting patterns of exclusion embedded in racialized spatial dynamics.

Consistent with previous studies (14, 59, 60), this study found a negative association between technology access and healthcare disparities. Individuals with better technology access were found to experience reduced healthcare disparities. This finding underscores the potential of technology in bridging healthcare gaps and improving access to care, especially for underserved populations, if, and only if, systemic barriers to access and utilization are dismantled. Telehealth specifically plays a crucial role in addressing healthcare disparities by providing access to medical care for individuals in underserved communities. It enables remote consultations, reduces wait times, and facilitates continuity of care, particularly for managing chronic diseases. Mobile health applications and electronic health records further support patient engagement by providing real-time health monitoring and improved access to medical information (47, 61). Thus, by providing remote access to healthcare services, technology can overcome geographical barriers and ensure timely delivery of care to individuals who may face challenges in accessing traditional healthcare facilities.

The study also found a positive association between technology access and health technology use, which is consistent with previous research (44, 62–64), suggesting that individuals with better technology access are more likely to engage with health technology tools. This finding emphasizes the importance of ensuring equitable technology access to facilitate health technology adoption and engagement among diverse populations. By leveraging health technology, individuals can actively manage their health, access educational resources, and engage in shared decision-making with healthcare providers, potentially leading to improved health outcomes (65).

Furthermore, the negative association between health technology use and healthcare disparities supports the notion that health technology can help reduce disparities in healthcare outcomes. Individuals who actively utilize health technology may benefit from improved health management, increased access to information, and better communication with healthcare providers (66, 67). These factors contribute to more equitable healthcare experiences and outcomes.

The interaction effect between SES and technology access on healthcare disparities highlights the importance of considering the interplay between socioeconomic factors and technology access. This finding suggests that addressing both SES disparities and technology access is crucial in reducing healthcare disparities. Government initiatives such as telehealth subsidies, broadband expansion programs, and Medicaid coverage for virtual healthcare services have the potential to improve access to telehealth for low-income populations (68). These policies have the potential to reduce financial and technological barriers that limit healthcare access for marginalized groups (69). Thus, strategies should focus not only on enhancing technology access but also on addressing underlying socioeconomic inequalities to achieve equitable healthcare outcomes for all individuals.

Regarding the mediation analysis, this study found that the joint mediation of technology access and health technology use in the association between SES and healthcare disparities was statistically significant, suggesting that health technology use plays a crucial role in reducing healthcare disparities among individuals with different socioeconomic backgrounds (70, 71).

As better technology access facilitates greater engagement with health technology, it is imperative that digital literacy programs are introduced to help individuals navigate telehealth platforms, use mobile health applications, use social media to seek accurate health information and access electronic health records. Community-based initiatives and partnerships between healthcare providers and technology organizations should be developed to enhance digital skills, particularly among older adults and low-income populations, ensuring equitable utilization of health technology, and consequently leading to improved healthcare outcomes.

Furthermore, our results suggest that health technology use partially mediates the relationship between technology access and healthcare disparities. Health technology use, such as health apps or remote monitoring devices, helps bridge gaps in healthcare access and improve patient engagement. By promoting health technology use, healthcare providers can enhance patient-provider communication, empower patients to take an active role in their care, and ultimately reduce disparities in healthcare outcomes.

Interestingly, while we observed a significant indirect effect of SES on health technology use through technology access, the mediation of health technology use in the relationship between SES and healthcare disparities was not statistically significant. This suggests that the effect of SES on healthcare disparities is not solely mediated by health technology use in the studied population. Other factors, such as access to healthcare facilities, provider-patient communication, and cultural competence in healthcare, may also contribute to healthcare disparities among individuals with varying SES levels.

## Conclusion

The results of this study suggest that socioeconomic status (SES), technology access, and healthcare technology use are all important factors that can influence healthcare disparity. Additionally, the results suggest that the effect of technology access on healthcare disparity is different for people with different SES levels. These findings have important implications for the design of policies and programs aimed at reducing healthcare disparities. Policies aimed at reducing healthcare disparities must prioritize racial justice, addressing digital redlining, technological exclusion, and the economic marginalization of Black communities. By leveraging health technology effectively, healthcare systems can work towards reducing disparities, enhancing patient engagement, and improving health outcomes for all populations. Future research and policy efforts should continue to explore innovative ways to maximize the potential of technology in promoting health equity and reducing healthcare disparities.

## Limitations

Despite the findings discussed above, there are a number of limitations to this study that should be considered in interpreting the results. This current study was conducted in the United States of America, and the results may not be generalizable to other countries. Additionally, the study relied on self-reported data, which may introduce response biases and rely on participants’ subjective perceptions. Future studies could employ objective measures and longitudinal designs to strengthen the validity of the findings. Furthermore, the study was cross-sectional, hence, the effect of these constructs on healthcare disparity in the long term is contestable. It is imperative to understand the effect of these constructs in the long term over time through a longitudinal study. Lastly, the technology access measures from HINTS Wave 6 captured only basic device ownership and general internet use, without details on internet quality or broadband access. Future research should consider more detailed measures of internet infrastructure.

## Data Availability

Publicly available datasets were analyzed in this study. This data can be found at: https://hints.cancer.gov/data/download-data.aspx#H6.

## References

[1] Brewer LC Fortuna KL Jones C Walker R Hayes SN Patten CA. Back to the future: achieving health equity through health informatics and digital health. JMIR Mhealth Uhealth. (2020) 8:e14512. doi: 10.2196/14512, PMID: 31934874 PMC6996775

[2] Koonin LM Hoots B Tsang CA Leroy Z Farris K Jolly B. Trends in the use of telehealth during the emergence of the COVID-19 pandemic - United States, January-March 2020. MMWR Morb Mortal Wkly Rep. (2020) 69:1595–9. doi: 10.15585/mmwr.mm6943a3, PMID: 33119561 PMC7641006

[3] Massey DS Denton NA. American apartheid: segregation and the making of the underclass In: Social stratification, class, race, and gender in sociological perspective. 2nd ed. Milton Park, Abingdon, Oxfordshire, United Kingdom: Routledge (2019). 660–70.

[4] Bonilla-Silva E. Rethinking racism: toward a structural interpretation. Am Sociol Rev. (1997) 62:465–80. doi: 10.2307/2657316

[5] Braveman PA Arkin E Proctor D Kauh T Holm N. Systemic and structural racism: definitions, examples, health damages, and approaches to dismantling. Health Aff. (2022) 41:171–8. doi: 10.1377/hlthaff.2021.01394, PMID: 35130057

[6] Williams DR Sternthal M. Understanding racial-ethnic disparities in health: sociological contributions. J Health Soc Behav. (2010) 51:S15–27. doi: 10.1177/0022146510383838, PMID: 20943580 PMC3468327

[7] Pollack CE Cubbin C Sania A Hayward M Vallone D Flaherty B. Do wealth disparities contribute to health disparities within racial/ethnic groups? J Epidemiol Community Health. (2013) 67:439–45. doi: 10.1136/jech-2012-200999, PMID: 23427209 PMC3686361

[8] Centers for Disease Control and Prevention. Community health and program services (CHAPS): health disparities among racial/ethnic populations. Clifton Road, Atlanta, GA, USA: US Department of Health and Human Services (2008).

[9] Huh J Koola J Contreras A Castillo A Ruiz M Tedone K. Consumer health informatics adoption among underserved populations: thinking beyond the digital divide. Yearb Med Inform. (2018) 27:146–55. doi: 10.1055/s-0038-1641217, PMID: 30157518 PMC6115231

[10] Jones CP. Levels of racism: a theoretic framework and a gardener's tale. Am J Public Health. (2000) 90:1212–5. doi: 10.2105/ajph.90.8.1212, PMID: 10936998 PMC1446334

[11] Hartman S. Lose your mother: a journey along the Atlantic slave route. New York, NY, USA: Macmillan (2008).

[12] Gilmore RW. What is to be done? Am Q. (2011) 63:245–65. doi: 10.1353/aq.2011.0020

[13] Connell CL Wang SC Crook LS Yadrick K. Barriers to healthcare seeking and provision among African American adults in the rural Mississippi Delta region: community and provider perspectives. J Community Health. (2019) 44:636–45. doi: 10.1007/s10900-019-00620-1, PMID: 30661152 PMC6612316

[14] Saeed SA Masters RM. Disparities in health care and the digital divide. Curr Psychiatry Rep. (2021) 23:61. doi: 10.1007/s11920-021-01274-4, PMID: 34297202 PMC8300069

[15] Badr J Motulsky A Denis J-L. Digital health technologies and inequalities: a scoping review of potential impacts and policy recommendations. Health Policy. (2024) 146:105122. doi: 10.1016/j.healthpol.2024.105122 38986333

[16] Ferryman K. Framing inequity in health technology: the digital divide, data bias, and racialization. Brooklyn, NY, USA: Just Tech Social Science Research Council (2022).

[17] Lorence DP Park H Fox S. Racial disparities in health information access: resilience of the digital divide. J Med Syst. (2006) 30:241–9. doi: 10.1007/s10916-005-9003-y, PMID: 16978003

[18] Simons RL Lei MK Klopack E Beach SRH Gibbons FX Philibert RA. The effects of social adversity, discrimination, and health risk behaviors on the accelerated aging of African Americans: further support for the weathering hypothesis. Soc Sci Med. (2021) 282:113169. doi: 10.1016/j.socscimed.2020.113169, PMID: 32690336 PMC7790841

[19] National Academies of Sciences, E. and Medicine. Communities in action: pathways to health equity. Washington (DC): National Academies Press (US) (2017). 28418632

[20] Adinkrah EK Cobb S Bazargan M. Delayed medical Care of Underserved Middle-Aged and Older African Americans with chronic disease during COVID-19 pandemic. Healthcare. (2023) 11:595. doi: 10.3390/healthcare11040595 36833128 PMC9956154

[21] Krawczyk CS Funkhouser E Kilby JM Vermund SH. Delayed access to HIV diagnosis and care: special concerns for the southern United States. AIDS Care. (2006) 18:35–44. doi: 10.1080/09540120600839280, PMID: 16938673 PMC2763374

[22] Lee J. The impact of health information technology on disparity of process of care. Int J Equity Health. (2015) 14:34. doi: 10.1186/s12939-015-0161-3, PMID: 25889891 PMC4392633

[23] Ward-Sutton C Williams NF Moore CL Manyibe EO. Assistive technology access and usage barriers among African Americans with disabilities: a review of the literature and policy. J Appl Rehabil Couns. (2020) 51:115–33. doi: 10.1891/JARC-D-19-00011, PMID: 33762779 PMC7985985

[24] Mavrou K Meletiou-Mavrotheris M Kärki A Sallinen M Hoogerwerf EJ. Opportunities and challenges related to ICT and ICT-AT use by people with disabilities: an explorative study into factors that impact on the digital divide. Technol Disabil. (2017) 29:63–75. doi: 10.3233/TAD-170174

[25] Subramony D. Reframing the digital divide within a ‘flat world’context: the McJulien lecture. Jacksonville, FL: Association for Educational Communications and Technology (AECT) International Convention (2014).

[26] Subramony DP. Understanding the complex dimensions of the digital divide: lessons learned in the Alaskan Arctic. J Negro Educ. (2007) 76:57–67.

[27] Mulia N Ye Y Greenfield TK Martinez P Patterson D Kerr WC. Inequitable access to general and behavioral healthcare in the US during the COVID-19 pandemic: a role for telehealth? Prev Med. (2023) 169:107426. doi: 10.1016/j.ypmed.2023.107426, PMID: 36709864 PMC9877144

[28] Heiney SP Donevant SB Arp Adams S Parker PD Chen H Levkoff S. A smartphone app for self-management of heart failure in older African Americans: feasibility and usability study. JMIR Aging. (2020) 3:e17142. doi: 10.2196/17142, PMID: 32242822 PMC7165307

[29] Hesse BW Moser RP Rutten LJF Kreps GL. The health information national trends survey: research from the baseline. J Health Commun. (2006) 11:vii–xvi. doi: 10.1080/10810730600692553, PMID: 16641070

[30] Nelson D Kreps G Hesse B Croyle R Willis G Arora N. The health information national trends survey (HINTS): development, design, and dissemination. J Health Commun. (2004) 9:443–60. doi: 10.1080/10810730490504233, PMID: 15513791

[31] Dillman DA Smyth JD Christian LM. Internet, phone, mail, and mixed-mode surveys: the tailored design method. New York: John Wiley & Sons (2014).

[32] Paccoud I Baumann M le Bihan E Pétré B Breinbauer M Böhme P. Socioeconomic and behavioural factors associated with access to and use of personal health records. BMC Med Inform Decis Mak. (2021) 21:18. doi: 10.1186/s12911-020-01383-9, PMID: 33435970 PMC7805047

[33] Scherer R Siddiq F. The relation between students’ socioeconomic status and ICT literacy: findings from a meta-analysis. Comput Educ. (2019) 138:13–32. doi: 10.1016/j.compedu.2019.04.011

[34] Dixit RR. Factors influencing Healthtech literacy: an empirical analysis of socioeconomic, demographic, technological, and health-related variables. Appl Res Artif Intell Cloud Comput. (2018) 1:23–37.

[35] Reddick CG Enriquez R Harris RJ Sharma B. Determinants of broadband access and affordability: an analysis of a community survey on the digital divide. Cities. (2020) 106:102904. doi: 10.1016/j.cities.2020.102904, PMID: 32921864 PMC7480260

[36] Gergen Barnett K Mishuris RG Williams CT Bragg A Semenya AM Baldwin M. Telehealth's double-edged sword: bridging or perpetuating health inequities? J Gen Intern Med. (2022) 37:2845–8. doi: 10.1007/s11606-022-07481-w, PMID: 35352272 PMC8963395

[37] de Buhr E Tannen A. Parental health literacy and health knowledge, behaviours and outcomes in children: a cross-sectional survey. BMC Public Health. (2020) 20:1096. doi: 10.1186/s12889-020-08881-5, PMID: 32660459 PMC7359277

[38] Jansen T Rademakers J Waverijn G Verheij R Osborne R Heijmans M. The role of health literacy in explaining the association between educational attainment and the use of out-of-hours primary care services in chronically ill people: a survey study. BMC Health Serv Res. (2018) 18:394. doi: 10.1186/s12913-018-3197-4, PMID: 29855365 PMC5984471

[39] Seljelid B Varsi C Solberg Nes L Øystese KA Børøsund E. Feasibility of a digital patient-provider communication intervention to support shared decision-making in chronic health care, InvolveMe: pilot study. JMIR Form Res. (2022) 6:e34738. doi: 10.2196/34738, PMID: 35389356 PMC9030980

[40] Spooner KK Salemi JL Salihu HM Zoorob RJ. eHealth patient-provider communication in the United States: interest, inequalities, and predictors. J Am Med Inform Assoc. (2016) 24:e18–27. doi: 10.1093/jamia/ocw087, PMID: 27497797 PMC7651920

[41] Lee HY Jin SW Henning-Smith C Lee J Lee J. Role of health literacy in health-related information-seeking behavior online: cross-sectional study. J Med Internet Res. (2021) 23:e14088. doi: 10.2196/14088, PMID: 33502332 PMC7875696

[42] Busse TS Nitsche J Kernebeck S Jux C Weitz J Ehlers JP. Approaches to improvement of digital health literacy (eHL) in the context of person-centered care. Int J Environ Res Public Health. (2022) 19:8309. doi: 10.3390/ijerph19148309, PMID: 35886158 PMC9316109

[43] Lee J Tak SH. Factors associated with eHealth literacy focusing on digital literacy components: a cross-sectional study of middle-aged adults in South Korea. Digit Health. (2022) 8:20552076221102765. doi: 10.1177/20552076221102765, PMID: 35615270 PMC9125061

[44] Borges do Nascimento IJ Abdulazeem HM Vasanthan LT Martinez EZ Zucoloto ML Østengaard L. The global effect of digital health technologies on health workers’ competencies and health workplace: an umbrella review of systematic reviews and lexical-based and sentence-based meta-analysis. Lancet Digit Health. (2023) 5:e534–44. doi: 10.1016/S2589-7500(23)00092-4 37507197 PMC10397356

[45] Eruchalu CN Pichardo MS Bharadwaj M Rodriguez CB Rodriguez JA Bergmark RW. The expanding digital divide: digital health access inequities during the COVID-19 pandemic in New York City. J Urban Health. (2021) 98:183–6. doi: 10.1007/s11524-020-00508-9, PMID: 33471281 PMC7816740

[46] Bates D. Health inequities and technology. J Health Care Poor Underserved. (2021) 32:viii–xii. doi: 10.1353/hpu.2021.004434120955

[47] LeeMNamS. Telehealth utilization among patients with chronic disease: insights from the 2022 health information national trends survey. J Telemed Telecare. (2024) 1:1357633X241289158. doi: 10.1177/1357633x241289158, PMID: 39501649

[48] Oluyede L Cochran AL Wolfe M Prunkl L McDonald N. Addressing transportation barriers to health care during the COVID-19 pandemic: perspectives of care coordinators. Transp Res A Policy Pract. (2022) 159:157–68. doi: 10.1016/j.tra.2022.03.010, PMID: 35283561 PMC8898700

[49] Park J-H Lee MJ Tsai MH Shih HJ Chang J. Rural, regional, racial disparities in telemedicine use during the COVID-19 pandemic among US adults: 2021 national health interview survey (NHIS). Patient Prefer Adherence. (2023) 17:3477–87. doi: 10.2147/PPA.S439437, PMID: 38143946 PMC10749101

[50] Larnyo E Dai B Larnyo A Nutakor JA Ampon-Wireko S Nkrumah ENK. Impact of actual use behavior of healthcare wearable devices on quality of life: a cross-sectional survey of people with dementia and their caregivers in Ghana. Healthcare. (2022) 10:275. doi: 10.3390/healthcare10020275, PMID: 35206890 PMC8872618

[51] Reinartz W Haenlein M Henseler J. An empirical comparison of the efficacy of covariance-based and variance-based SEM. Int J Res Mark. (2009) 26:332–44. doi: 10.1016/j.ijresmar.2009.08.001

[52] Ringle CM Wende S Becker J-M. SmartPLS 4. Oststeinbek: SmartPLS (2022). Available at: https://www.smartpls.com/

[53] Hair JF Hult GTM Ringle CM Sarstedt M Danks NP Ray S. Evaluation of formative measurement models In: Hair JF Jr Hult GTM Ringle CM Sarstedt M Danks NP Ray S, editors. Partial least squares structural equation modeling (PLS-SEM) using R: A workbook. Cham: Springer International Publishing (2021). 91–113.

[54] Hair JF Sarstedt M Ringle CM. Rethinking some of the rethinking of partial least squares. Eur J Mark. (2019) 53:566–84. doi: 10.1108/EJM-10-2018-0665

[55] Hair JF Risher JJ Sarstedt M Ringle CM. When to use and how to report the results of PLS-SEM. Eur Bus Rev. (2019) 31:2–24. doi: 10.1108/EBR-11-2018-0203

[56] Kline R.B., Principles and practice of structural equation modeling (3rd ed). New York, NY: Guilford, (2011). 14: p. 1497–1513.

[57] Hu LT Bentler PM. Cutoff criteria for fit indexes in covariance structure analysis: conventional criteria versus new alternatives. Struct Equ Model Multidiscip J. (1999) 6:1–55. doi: 10.1080/10705519909540118

[58] Pick JB Azari R. Global digital divide: influence of socioeconomic, governmental, and accessibility factors on information technology. Inf Technol Dev. (2008) 14:91–115. doi: 10.1002/itdj.20095

[59] Barnett ML Ray KN Souza J Mehrotra A. Trends in telemedicine use in a large commercially insured population, 2005-2017. JAMA. (2018) 320:2147–9. doi: 10.1001/jama.2018.12354, PMID: 30480716 PMC6349464

[60] Haimi M. The tragic paradoxical effect of telemedicine on healthcare disparities- a time for redemption: a narrative review. BMC Med Inform Decis Mak. (2023) 23:95. doi: 10.1186/s12911-023-02194-4, PMID: 37193960 PMC10186294

[61] Bouabida K Lebouché B Pomey M-P. Telehealth and COVID-19 pandemic: an overview of the telehealth use, advantages, challenges, and opportunities during COVID-19 pandemic. Healthcare. (2022) 10:2293. doi: 10.3390/healthcare10112293, PMID: 36421617 PMC9690761

[62] Sawesi S Rashrash M Phalakornkule K Carpenter JS Jones JF. The impact of information technology on patient engagement and health behavior change: a systematic review of the literature. JMIR Med Inform. (2016) 4:e1. doi: 10.2196/medinform.4514, PMID: 26795082 PMC4742621

[63] Kruse CS Beane A. Health information technology continues to show positive effect on medical outcomes: systematic review. J Med Internet Res. (2018) 20:e41. doi: 10.2196/jmir.8793, PMID: 29402759 PMC5818676

[64] Nielsen P Sahay S. A critical review of the role of technology and context in digital health research. Digit Health. (2022) 8:20552076221109554. doi: 10.1177/20552076221109554, PMID: 35769359 PMC9234838

[65] Chatterjee A Shahaab A Gerdes MW Martinez S Khatiwada P. Chapter 22 - leveraging technology for healthcare and retaining access to personal health data to enhance personal health and well-being In: Bhattacharyya S Dutta P Samanta D Mukherjee A Pan I, editors. Recent trends in computational intelligence enabled research: Academic Press (2021). 367–76.

[66] van Veen T Binz S Muminovic M Chaudhry K Rose K Calo S. Potential of mobile health technology to reduce health disparities in underserved communities. West J Emerg Med. (2019) 20:799–802. doi: 10.5811/westjem.2019.6.41911, PMID: 31539337 PMC6754190

[67] ElKefi S Asan O. How technology impacts communication between cancer patients and their health care providers: a systematic literature review. Int J Med Inform. (2021) 149:104430. doi: 10.1016/j.ijmedinf.2021.104430, PMID: 33684711 PMC8131252

[68] Horrigan JB Whitacre BE Galperin H. Understanding uptake in demand-side broadband subsidy programs: the affordable connectivity program case. Telecommun Policy. (2024) 48:102812. doi: 10.1016/j.telpol.2024.102812

[69] Dullet NW Geraghty EM Kaufman T Kissee JL King J Dharmar M. Impact of a university-based outpatient telemedicine program on time savings, travel costs, and environmental pollutants. Value Health. (2017) 20:542–6. doi: 10.1016/j.jval.2017.01.014, PMID: 28407995

[70] Zhang X Hailu B Tabor DC Gold R Sayre MH Sim I. Role of health information technology in addressing health disparities: patient, clinician, and system perspectives. Med Care. (2019) 57:S115–20. doi: 10.1097/mlr.0000000000001092, PMID: 31095049 PMC6589829

[71] Veinot TC Ancker JS Bakken S. Health informatics and health equity: improving our reach and impact. J Am Med Inform Assoc. (2019) 26:689–95. doi: 10.1093/jamia/ocz132, PMID: 31411692 PMC7647232

